# Extracellular Matrix Influences Gene Expression and Differentiation of Mouse Trophoblast Stem Cells

**DOI:** 10.1089/scd.2022.0290

**Published:** 2023-10-03

**Authors:** Bryony V. Natale, Ramie Kotadia, Katarina Gustin, Anirudha Harihara, Sarah Min, Michael J. Kreisman, Kellie M. Breen, David R.C. Natale

**Affiliations:** Department of Obstetrics, Gynecology, and Reproductive Sciences, University of California San Diego, La Jolla, California, USA.

**Keywords:** trophoblast, trophoblast stem cell, extracellular matrix, differentiation, mouse

## Abstract

Trophoblast stem (TS) cells were first isolated from the mouse placenta; however, little is known about their maintenance and niche in vivo. TS cells, like other stem cells, have a unique microenvironment in which the extracellular matrix (ECM) is a component. Placental pathology is associated with ECM change. However, how these changes and the individual ECM components impact the maintenance or differentiation of TS cells has not been established. This study identified which ECM component(s) maintain the greatest expression of markers associated with undifferentiated mouse trophoblast stem (mTS) cells and which alter the profile of markers of differentiation based on mRNA analysis. mTS cells cultured on individual ECM components and subsequent quantitative polymerase chain reaction analysis revealed that laminin promoted the expression of markers associated with undifferentiated TS cells, fibronectin promoted gene expression associated with syncytiotrophoblast (SynT) layer II cells, and collagen IV promoted the expression of genes associated with differentiated trophoblast. To investigate whether pathological placental ECM influenced the expression of genes associated with different trophoblast subtypes, the mouse model of streptozotocin (STZ)-induced pancreatic β cell ablation and diabetes was used. Female mice administered STZ (blood glucose ≥300 mg/dL) or control (blood glucose ≤150 mg/dL) were mated. Placental pathology at embryonic day (E)14.5 was confirmed with reduced fetal blood space area, reduced expression of the pericyte marker αSMA, and decreased expression of ECM proteins. mTS cells cultured on ECM isolated from STZ placenta were associated with reduced expression of undifferentiated mTS markers and increased expression of genes associated with terminally differentiated trophoblast [*Gcm-1* and *SynA* (SynT) and junctional zone *Tpbpa* and *Prl2c2*]. Altogether, these results support the value of using ECM isolated from the placenta as a tool for understanding trophoblast contribution to placental pathology.

## Introduction

Stem cells contribute to the maintenance and regeneration of tissue. In the placenta, trophoblast stem (TS) cells are responsible for the generation of differentiated trophoblast cells that contribute to placental function [1]. TS cells were first isolated from the mouse placenta [1], and their study informed much of what was known about trophoblast proliferation and differentiation before the identification and isolation of the human TS cell [2]; however, little is known about their maintenance in vivo. TS cells, like other stem cells, have a unique microenvironment or niche. One essential component of this niche is the extracellular matrix (ECM), which can regulate cell behavior through biophysical, mechanical, and biochemical properties.

Cells constantly rebuild and remodel the ECM through synthesis, degradation, reassembly, and chemical modification. Tissue injury can lead to dysregulated ECM remodeling, which is associated with pathological conditions and can exacerbate disease progression [3]. It is well established that individual ECM components and ECM substrates impact TS cell behavior [2,4–6]. Placental ECM comprises different components, largely collagen IV, laminin, and fibronectin. In the human placenta, the ECM of the villi is characterized by the presence of laminin, while closer to the maternal interface, it contains more fibronectin. In the murine placenta, the labyrinth ECM contains collagen, laminin, and fibronectin [7–12]. In placental pathology, in both human and animal models, change in ECM components have been reported [9,13–18]. The mechanobiology of trophoblast cells and their response to ECM is well explored in the context of trophoblast invasion and syncytiotrophoblast (SynT) fusion [4,19] but less so in the context of the TS cell niche.

When human TS cells were first isolated, they were cultured on collagen IV [2], while mouse trophoblast stem (mTS) cells are commonly grown on Matrigel, which is primarily laminin and collagen IV [20–22] or in the absence of ECM on tissue culture plastic, with isolated studies using individual ECM components. With increased interest in the development of engineered biomaterials, which may better mimic the in vivo characteristics of stem cell microenvironments, it is important to evaluate how the placental microenvironment impacts TS cells. Comparative analysis of the effect of ECM on the maintenance of undifferentiated trophoblast cells, together with their differentiation potential, is limited. As the ability for a stem cell to seed its niche is critical to the maintenance of a stem cell pool for long-term self-renewal [23,24], this information would be critical to understanding how altered ECM in placental pathology may contribute to the presence and/or maintenance of undifferentiated trophoblast.

Thus, to further understand how ECM components affect the proliferation and differentiation profile of mTS cells in culture, this study aimed to identify which ECM component kept TS cells in their least differentiated state and whether different components were optimal for differentiation to specific trophoblast subtypes, based on mRNA analysis. With altered undifferentiated (proliferative) trophoblast populations [10,25] and altered ECM profiles reported in placental pathology, this study further aimed to identify whether ECM isolated from mid-late gestation pathological mouse placentae had the capacity to alter the expression of markers associated with undifferentiated and differentiated trophoblast.

## Materials and Methods

### mTS cell culture

Cell lines (previously derived in our laboratory from E3.5 blastocyst) [26] were cultured in 5% CO_2_ in humidified air at 37°C in RPMI medium (Life Technologies) supplemented with 20% fetal bovine serum (Life Technologies), 1 mM sodium pyruvate (Life Technologies), 50 μg/mL penicillin/streptomycin (Life Technologies), 25 ng/mL FGF4 (R&D), 10 ng/mL recombinant Activin A (R&D), 1 μg/mL heparin (Sigma), and 55 μM β-mercaptoethanol (Life Technologies) to maintain an undifferentiated, proliferative state, as previously described [27]. To differentiate cells, medium without FGF4, Activin A, and heparin was used. TS cells were cultured for 2 days in proliferating conditions as an undifferentiated control (referred to as day 0 due to the 0 days of differentiation) and for 2, 4, and 6 days in differentiating TS cell conditions [26,27]. In all experiments, cells were seeded at a density of 1.4 × 10^3^ cells/cm^2^.

Due to the heterogeneous nature of mTS cell cultures, genes of both undifferentiated TS cells and differentiated trophoblast were evaluated in both proliferating (+FGF4) and differentiating (−FGF4) conditions. The ECM component-treated plastic included commercially available collagen IV-, fibronectin-, and laminin-coated plastic six-well plates (Corning).

### RNA, cDNA, and quantitative reverse transcription–polymerase chain reaction

As previously published [26,28], total RNA was isolated from TS cell cultures (Aurum micro spin columns; Bio-Rad) and placentae (Direct-zol RNA miniprep kit; Zymo Research) according to the manufacturer's protocols, including the DNase digestion step. mRNA expression was evaluated by quantitative reverse transcription–polymerase chain reaction (qRT-PCR), using the SYBR green method according to the manufacturer's protocol (Bio-Rad) and as previously described [10,26]. RT used 1 μg of total RNA (iScript RT Kit for SYBR Green Supermix; Bio-Rad). qRT-PCR reactions (iTaq Universal SYBR Green Supermix; Bio-Rad) were performed with a Bio-Rad CFX96 thermocycler with Bio-Rad primers (Supplementary Table S1). The cycle threshold was set so that exponential increases in amplification were approximately level between all samples. For all placental analyses, biological replicates included six placentae (two placentae from each of three dams) and for tissue culture, biological replicates included experiments performed in triplicate (*n* = 3/treatment/days in culture).

Data were analyzed using the comparative cycle threshold (ΔΔCt) method, as previously described [29], using *Ppia* and *Ywhaz* (Supplementary Table S1) as reference genes. cDNA samples were run in triplicate (technical replicate), and the relative quantity of the target genes was calculated using the formula 2^−ΔΔCT^ using the Bio-Rad Relative Expression Software Tool. Data are presented as a fold change in gene expression normalized to two validated reference genes relative to the control.

### Mouse model of unmanaged maternal hyperglycemia

Adult female C57BL/6 mice (12 weeks; Envigo) were housed two animals per cage under standard conditions with 12-h light/12-h dark cycle with lights on at 06:00 h. Animals had ad libitum access to water and Harlan irradiated chow (#2920X) at a University of California, San Diego vivarium.

Streptozotocin (STZ) treatment to induce hyperglycemia was initiated 2 weeks before mating. For three consecutive days, females received either a single intraperitoneal injection of STZ (75 mg/kg; diluted in 0.1 M citrate buffer, pH 4.5; treatment; Sigma) or a vehicle (VEH) control injection of the equivalent volume of 0.1 M citrate buffer, pH 4.5. Blood glucose was assessed 7- and 14-days following treatment using a Glucometer (OneTouch Ultra 2) by tail sampling. Blood glucose was confirmed in VEH-treated animals (<150 mg/dL) and STZ-treated animals (>300 mg/dL). Two weeks posttreatment, females were weighed and mated with untreated C57BL/6 males, resulting in pregnancies exposed to elevated glucose from conception (*n* = 7; STZ dams and *n* = 5 VEH dams). Successful mating was confirmed by a copulatory plug the morning after mating and considered E0.5.

Placentae from these pregnancies are herein referred to as STZ or VEH placentae. All mouse work was conducted as per University of California San Diego guidelines for the use of animal models in research, with user protocols and experiments reviewed and approved by the Institutional Animal Care and Use Committee at the University of California San Diego.

### Placenta collection and preparation

Pregnant dams were euthanized by cervical dislocation at E14.5. Placentae were dissected and collected for histological assessment and ECM isolation. Placentae for histological analysis were bisected and fixed in 4% paraformaldehyde/phosphate-buffered saline (PBS) overnight, washed in PBS, dehydrated through an ethanol series, and paraffin embedded as previously described [10,27,30,31]. Paraffin blocks were sectioned using a Leica microtome (5–7 μm) for analysis. E14.5 placentae for ECM isolation were collected and processed as described below.

### Immunohistochemistry

Immunohistochemistry (IHC) was performed as per the published protocol (ImmPRESS Horse Anti-Rabbit IgG Kit; Vector Laboratories). Briefly, antigen retrieval (Citra Buffer; BioGenex) was performed in a 2100-Retriever (Electron Microscopy Science). Primary antibodies were diluted in kit-supplied, ready-to-use horse serum and visualized using Dako DAB or Vector VIP according to the manufacturer's protocol (Dako; Vector Laboratories, respectively) with Hematoxylin or Nuclear Fast Red counterstain (Gills #2; Sigma; Vector Laboratories, respectively). All IHC was conducted with their respective negative controls (omission of primary antibody). Antibodies were used at the following dilutions: anti-Ki67, 1:100 (ab16667; Abcam); anti-αSMA, 1:200 (ab5694; Abcam); anti-Laminin, 1:200 (ab11575; Abcam); anti-Collagen, 1:200 (ab6586; Abcam); anti-Fibronectin, 1:200 (ab23750; Abcam) and anti-MCT4, 1:400 (ab3314P; EMD Millipore).

### Histological staining

Endogenous alkaline phosphatase (AP) staining was used to identify the maternal blood spaces, while isolectin B4 (L5391; Sigma) staining was used to identify the basement membrane of the fetal capillaries (herein referred to as fetal blood space) in the labyrinth layer of the placenta, as previously described [32,33].

### Histological evaluation

Placenta tissue for all staining included randomly selected sections from the center of the placenta. After staining, placentae were imaged (EVOS, Life Technologies) and staining was measured using ImageJ analysis software [34] (image and measurement details below). A single observer, blinded to experimental conditions at the time of the assessments, performed all assessments/quantification and a second blinded observer confirmed the results.

#### Placenta layers

Analysis of placental layers used images of isolectin-stained placentae to identify the labyrinth (isolectin positive), with the junctional zone layer, the isolectin-negative region between the labyrinth layer and the parietal trophoblast giant cells (TGCs) that separate the junctional zone from the maternal decidua. The labyrinth, junctional zone, and total area of the placentae were manually traced, with each layer presented as a percentage of the total placenta area [10,11,33,35]. Periodic acid–Schiff (PAS) stain was used for placental histological representation, as per the manufacturer's protocol (Sigma).

#### Labyrinth-specific analysis

Proliferation assessment (Ki67 IHC; 400 × magnification; stained nuclei considered a single-positive cell) [10,11] was performed on three nonoverlapping labyrinth-specific images/placenta and is presented as Ki67^+^ cells/field-of-view (FOV; 100 × magnification images used as Ki67 histological representative images). Labyrinth-specific FOV images excluded junctional zone and fetal membranes, with one image taken in the center of the labyrinth and the remaining images taken midway between the center and the outside of the region (images with a canal artery through the image were excluded).

αSMA [10,11], laminin [11,12], collagen [11,12], fibronectin [12], and MCT4 IHC (400 × magnification) staining was quantified using three to five nonoverlapping labyrinth-specific images/placenta with stained area presented as a percentage of the FOV. Labyrinth fetal capillary and maternal blood sinusoid space assessment [10,35–37] used three to five nonoverlapping labyrinth-specific 400 × images/placenta from AP-stained sections, where maternal blood sinusoids were identified by AP-positive staining, and an absence of AP staining identified fetal capillaries.

All blood spaces were manually outlined, and the area and perimeter of each space were measured, with findings presented as a perimeter-to-area ratio.

### Isolation of placental ECM and coating of tissue culture plastic

Isolation of placental ECM was performed using previously described methods [38]. Briefly, ECM was isolated from 16 to 20 bisected E14.5 placentae (dissected from 2 dams/treatment group) for STZ- and VEH-treated pregnancies.

Placentae were trimmed of fetal membranes and maternal decidua so that isolated ECM was only from the fetal-derived placenta. Placentae were rinsed with deionized water (30 min) to remove blood and debris, with all STZ placentae pooled and all VEH placentae pooled before decellularization in 1% sodium dodecyl sulfate (SDS)/PBS solution for 5 days (RT; gentle agitation). The detergent was removed with 2 × (5 min) rinses plus an overnight rinse in deionized water. Decellularized ECM was frozen, lyophilized overnight, and milled to a fine powder using a mortar and pestle. After milling, ECM was digested in pepsin/HCL solution (1 mg/mL Pepsin in 0.1 M HCl) for 2 days and diluted in 0.1 M acetic acid to a concentration of 10 mg/mL. The thickness of ECM has been shown to affect mechanical stress and differentiation [19,39]. Therefore, to ensure that the analysis was based only on the isolated ECM and not the additional effect of mechanical stress, the tissue culture plates were equally coated with ECM.

ECM was diluted in sterile PBS to a final concentration of 620 μg/mL (500 μL/well −12-well plates and 1 mL/well −6-well plates; used at 65–77 μg/cm^2^). Once coated, plates were baked at 37°C for an hour and rinsed with sterile 1 × PBS before plating cells. Cells were seeded at 1.4 × 10^3^ cells/cm^2^ and were cultured for 0, 2, 4, and 6 days in the presence and absence of growth factors as described above. Experiments were performed in triplicate as biological replicates (*n* = 3). The analysis included histological analysis (H&E of day 0 cells to show undifferentiated colony-forming status [26,40]; H&E of day 6 cells to show differentiated cell morphology [26] and PAS stain as evidence of differentiation to glycogen trophoblast cells (GlyT) based on glycogen accumulation [26]). Together with a qRT-PCR panel for undifferentiated TS cells and differentiated trophoblast populations, these assays served as evidence of an undifferentiated versus differentiated status of the TS cells.

### Statistical analysis

Analysis was performed using Prism 9 software. Specifically, qPCR results from TS cells cultured on ECM component tissue culture were analyzed by two-way ANOVA using Tukey's multiple comparisons test to identify the main effects: (1) across all treatment groups, which day(s) of culture had peak gene expression and (2) across all days of culture, which treatment(s) yielded the greatest gene expression. Following the main effect analysis, further simple effect analysis by Tukey's multiple comparisons test was performed to identify significance within the ECM treatment groups on the day of peak expression identified in the main effect analysis. Based on the rationale that the experimental questions were best answered by multiple comparison tests [41–43], results and figures identify significant multiple comparison findings in two-way ANOVA tables. The qPCR results from TS cells cultured on placental isolated ECM were analyzed by two-way ANOVA using Šídák's multiple comparisons with a single pooled variance with multiplicity-adjusted *P* value for each comparison.

qPCR results evaluating the expression of genes involved in cell/cell interaction and cell junctions in day 0 mTS cells grown on isolated placental ECM were analyzed using multiple *t*-test analysis (corrected for multiple comparisons using the Holm–Šídák method). For all two-way ANOVA analyses, significant interactions between the factors are reported (a significant interaction is when the difference in the means of the response between the levels of one factor is not the same across all levels of another factor). Significance was identified as a *P* value or adjusted *P* value ≤0.05, with all data expressed as mean ± standard error of the mean (SEM).

Dam glucose (mg/dL) and dam weight (g) were analyzed by two-way ANOVA, with day posttreatment (D16/D0) and treatment (VEH/STZ) as factors. Post hoc analysis using Šídák's multiple comparisons with a single pooled variance with multiplicity-adjusted *P* value for each comparison was performed. Placenta histological quantifications (percentage or cell number) and qPCR results were analyzed by unpaired *t*-test. Significance was identified as a *P* value or adjusted *P* value ≤0.05, with data expressed as mean ± SEM.

## Results

### Individual ECM components promote maintenance of different trophoblast subtypes

TS cells grown for 2 days in proliferating conditions (day 0; undifferentiated control) and cells grown in differentiating conditions (2, 4, and 6 days) were used to assess whether the ECM components (collagen IV, fibronectin, and laminin) would alter the expression of mRNAs characteristic of undifferentiated and differentiated trophoblast. Results identified main and simple effects (summary table associated with each figure includes overall ANOVA results and post hoc analysis).

#### Genes associated with undifferentiated mTS cells

**FIG. 1.**
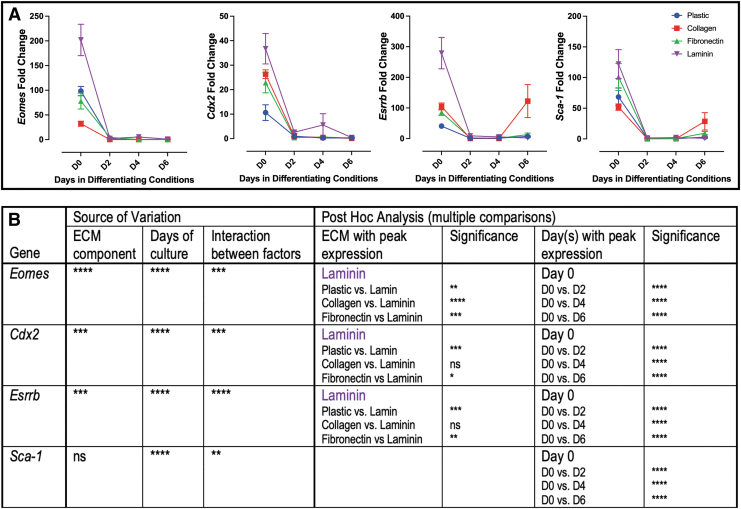
Markers of undifferentiated mTS cells peak on laminin. **(A)** qPCR analysis of markers of undifferentiated mTS cells peak on day 0; *Eomes*, *Cdx2*, and *Esrrb* peak on laminin ECM. **(B)** Two-way ANOVA table identifying the source of variation (ECM, days in culture and interaction between factors); post hoc analysis identified specific ECM with peak expression and specific days in culture by Tukey's multiple comparisons test. Bold font ECM or days in culture identifies a parameter with post hoc significance in 3/3 comparisons; regular font identified a parameter with post hoc significance in 2/3 comparisons; no conclusion was made with significance in less than 2 comparisons. *Stars* indicate statistical significance, *P ≤ 0.05, **P ≤ 0.01, ***P ≤ 0.001, ****P ≤ 0.0005. Error bars are SEM. ECM, extracellular matrix; mTS, mouse trophoblast stem; n/s, no significance; qPCR, quantitative polymerase chain reaction; SEM, standard error of the mean.

mTS cell markers *Eomes*, *Cdx2*, and *Esrrb* had peak expression on day 0 with overall peak expression on laminin (Fig. 1). *Sca1* expression similarly peaked on day 0; however, no ECM component made a significant difference in the overall level of expression (Fig. 1).

#### Genes associated with labyrinth trophoblast

**FIG. 2.**
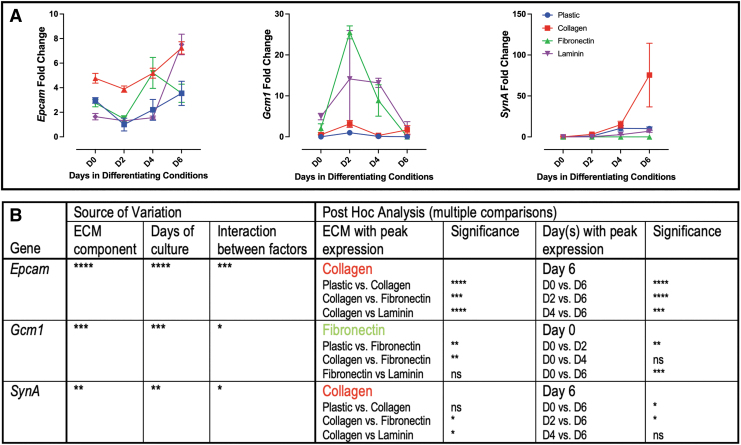
Markers of labyrinth trophoblast grown on individual ECM components. **(A)** qPCR analysis of markers of labyrinth trophoblast. **(B)** Two-way ANOVA table identifying the source of variation (ECM, days in culture and interaction between factors); post hoc analysis identified specific ECM with peak expression and specific days in culture by Tukey's multiple comparisons test. *Bold* font ECM or days in culture identifies a parameter with post hoc significance in 3/3 comparisons; *regular* font identified a parameter with post hoc significance in 2/3 comparisons; no conclusion made with significance in less than 2 comparisons. *Stars* indicate statistical significance, *P ≤ 0.05, **P ≤ 0.01, ***P ≤ 0.001, ****P ≤ 0.0001. Error bars are SEM.

Labyrinth progenitor marker *Epcam* peaked on day 6 with overall expression increased on collagen (Fig. 2). SynT trophoblast marker *Gcm-1* peaked on day 2 with the greatest expression on fibronectin, while *SynA,* like *Epcam,* peaked on day 6 with increased expression on collagen (Fig. 2).

#### Genes associated with junctional zone trophoblast

**FIG. 3.**
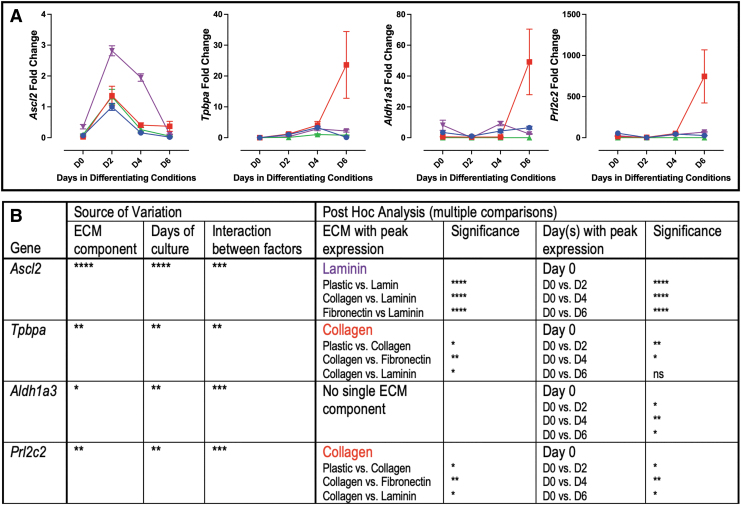
Markers of junctional zone trophoblast grown on individual ECM components. **(A)** qPCR analysis of markers of junctional zone trophoblast. **(B)** Two-way ANOVA table identifying the source of variation (ECM, days in culture, and interaction between factors); post hoc analysis identified specific ECM with peak expression and specific days in culture by Tukey's multiple comparisons test. *Bold* font ECM or days in culture identifies a parameter with post hoc significance in 3/3 comparisons; regular font identified a parameter with post hoc significance in 2/3 comparisons; no conclusion made with significance in less than 2 comparisons. *Stars* indicate statistical significance, *P ≤ 0.05, **P ≤ 0.01, ***P ≤ 0.001, ****P ≤ 0.0001. Error bars are SEM.

Junctional zone progenitor marker *Ascl2* expression peaked on day 2, with the greatest expression on laminin (Fig. 3). *Tpbpa* and *Prl2c2* both had expression peak on day 6, with collagen having the greatest influence on expression (Fig. 3). *Aldh1a3* similarly had expression peak on day 6; however, no single ECM component significantly influenced expression (Fig. 3).

### STZ-induced diabetes results in pregnancies associated with placental pathology and an altered ECM profile

**FIG. 4.**
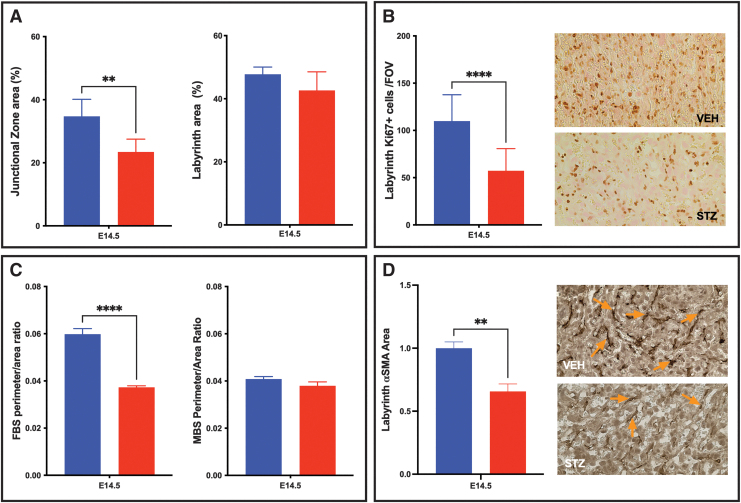
Identification of placental pathology in STZ-induced diabetic placentae. **(A)** Junctional Zone area as a percentage of total placental area is reduced in the STZ placentae; labyrinth area is not altered. **(B)** Total labyrinth Ki67 proliferation at E14.5 is reduced; representative histological images of placenta labyrinth. **(C)** Fetal and maternal blood space analysis included assessment of blood space perimeter to area ratio at E14.5. FBS perimeter to area ratio is reduced in STZ placentae. **(D)** Labyrinth pericyte area reduced in STZ placentae as identified by αSMA staining, analysis at E14.5; representative histological images (example of αSMA staining *circled* in *orange* with an *orange arrow* identifying stain, remaining *orange arrows* and *dark brown* stain identify αSMA fetal blood space-associated pericytes). *t*-Test analysis was performed to identify significance. *Stars* indicate statistical significance between the treatment groups, **P ≤ 0.01, ****P ≤ 0.0001. Error bars are SEM. Image magnification in **(B, D)** = 400 × (*n* ≥ 6 placentae per treatment). FBS, fetal bovine serum; STZ, streptozotocin.

As individual ECM components altered TS cell differentiation and changes in the expression of ECM proteins have been reported in placental pathology, the STZ model of diabetes was used to assess whether this model was associated with changes in placental ECM. Dams treated with STZ versus VEH control had threefold elevated blood glucose at mating (560 ± 14.83 mg/dL vs. 131 ± 4.05 mg/dL; data not shown), confirming successful targeting of pancreatic β cells by STZ, as previously reported [44–46]. Placentae from the resulting pregnancies were analyzed for placental pathology (Fig. 4). The placentae isolated from the STZ pregnancies had a smaller junctional zone when compared with the VEH control placentae (Fig. 4A and Supplementary Fig. S1), and while there was no change in the size of the labyrinth in the STZ placentae (Fig. 4A), there was a reduction in labyrinth proliferation (identified by Ki67 staining; Fig. 4B), fetal blood space perimeter to area ratio (Fig. 4C; no change in maternal blood space perimeter to area ratio), and pericyte coverage of the fetal-derived labyrinth vessels (identified by αSMA staining; Fig. 4D).

**FIG. 5.**
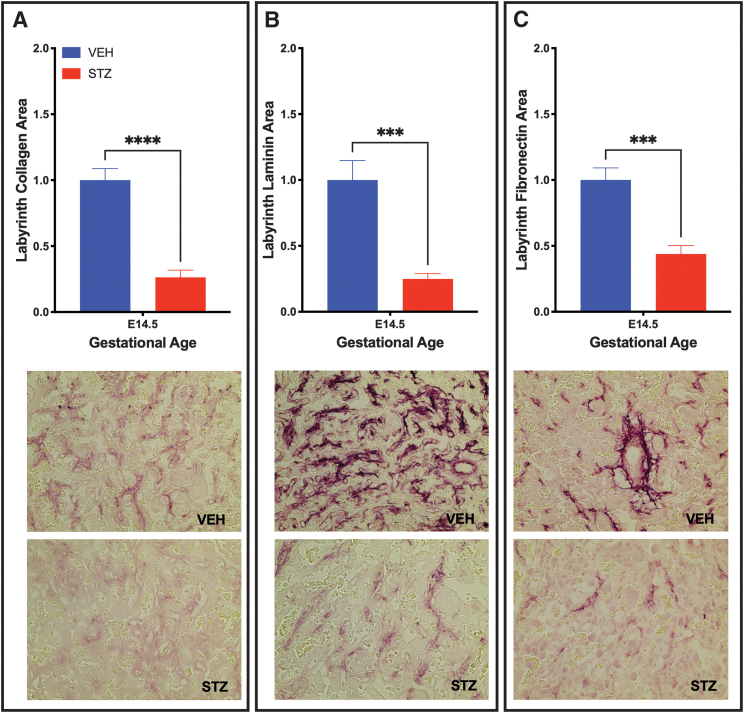
Placentae from STZ-induced diabetic placenta have reduced ECM components. Labyrinth ECM was altered in STZ placentae as identified by Collagen **(A)**, Laminin **(B)**, and Fibronectin **(C)** at E14.5 with representative histological images. *Stars* indicate statistical significance between the treatment groups, ***P ≤ 0.001, ****P ≤ 0.0001. Error bars are SEM. Image magnification = 400 × (*n* ≥ 6 placentae per treatment).

The pathology in the STZ placentae was further associated with a change in the ECM components (collagen IV, laminin, and fibronectin), with labyrinth staining of each of the three components reduced by greater than half when compared with the VEH control placentae (Fig. 5).

### mTS cells grown on ECM isolated from STZ-induced diabetic placentae have an altered differentiation profile

With pathology confirmed, E14.5 placental ECM was isolated from pooled placentae from each treatment group (VEH and STZ) to assess whether tissue culture plastic treated with the isolated placental ECM from the STZ placentae would alter the trophoblast gene profile of the differentiating TS cells when compared with TS cells grown on placental ECM from the healthy VEH placentae.

#### Genes associated with undifferentiated mTS cells

**FIG. 6.**
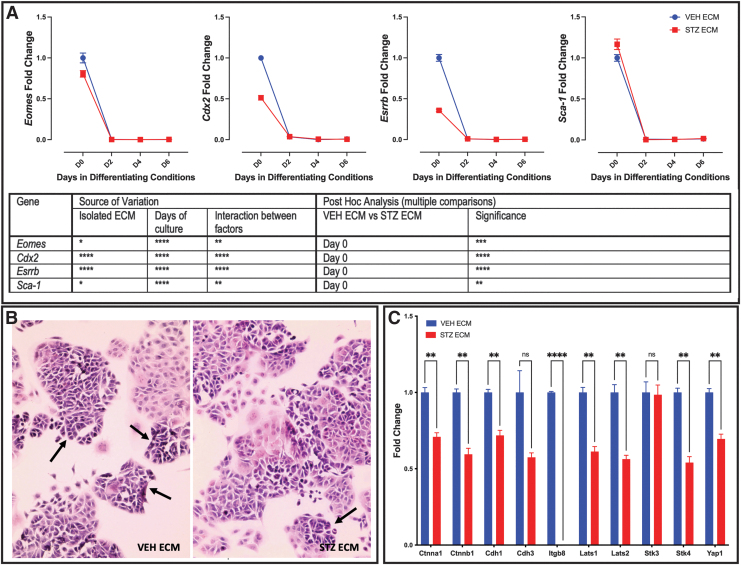
Pathological ECM alters expression of markers of undifferentiated mTS cells. **(A)** qPCR analysis of markers of undifferentiated mTS cells identified peak expression of all markers on day 0; *Eomes*, *Cdx2*, and *Esrrb* expression was reduced on STZ ECM, while *Sca-1* expression was increased on STZ ECM; two-way ANOVA table identifying the source of variation (ECM, days in culture, and interaction between factors by *row*); post hoc analysis using Šídák's multiple comparisons test identified which day expression was significantly altered by pathological ECM. **(B)** H&E stain of day 0 mTS cells cultured on placental isolated ECM; *arrows* identify mTS colonies with tight compact morphology; 200 × magnification **(C)** mTS cells grown on STZ ECM have reduced expression of markers associated with cell junction and cell/cell interaction. Multiple *t*-test analysis (corrected for multiple comparisons using the Holm–Šídák method) performed to identify significance with stars indicating statistical significance, *P ≤ 0.05, **P ≤ 0.01, ***P ≤ 0.001, ****P ≤ 0.0001. Error bars are SEM. H&E, Hematoxylin and Eosin.

mTS cell markers *Eomes*, *Cdx2*, *Esrrb*, and Sca1 had peak expression on day 0 (Fig. 6A), matching the day of peak expression to cells grown on individual ECM (compare Fig. 6A with Fig. 1). Post hoc analysis identified that mTS cells grown on ECM isolated from STZ pregnancies had reduced expression of *Eomes*, *Cdx2*, and *Esrrb* on day 0 when compared with those grown on VEH ECM (Fig. 6A).

Conversely, *Sca1* expression was increased on day 0 in cells grown on ECM isolated from STZ pregnancies and similarly peaked on day 0, when compared with those grown on VEH ECM (Fig. 6A). Histological staining confirmed that the day 0 cells maintained the colony-forming characteristics of undifferentiated mTS cells [26,47–50] when grown on isolated VEH and STZ placental ECM (Fig. 6B). mTS cells proliferate indefinitely. However, they are highly heterogeneous, with the population made up of different types of undifferentiated mTS cells as well as those that have differentiated [48]. Type 1 mTS cells are responsible for colony formation and present as small compact cells with tight cell junctions. Types 2–4 have loose colonies and flatter morphology with reduced expression of cell junction markers [48].

The morphology of the mTS cells grown on isolated VEH and STZ ECM were slightly different, with more colonies with the small compact morphology and tight cell junctions in the mTS cells grown on VEH ECM and fewer colonies with the small compact morphology (Fig. 6B arrows) and more loose colonies with flatter morphology on STZ ECM (Fig. 6B). Supporting the mTS colonies on the STZ ECM having loose colonies, qPCR analysis identified that 8 out of 10 markers associated with cell/cell interaction and cell junctions were decreased in the mTS cells grown on the isolated STZ ECM (Fig. 6C). With the decreased expression of *Eomes*, *Cdx2*, and *Esrrb,* altered morphology and changes in expression of cell/cell interaction and cell junctions, it appeared that the STZ ECM might promote differentiation.

#### Genes associated with labyrinth trophoblast

**FIG. 7.**
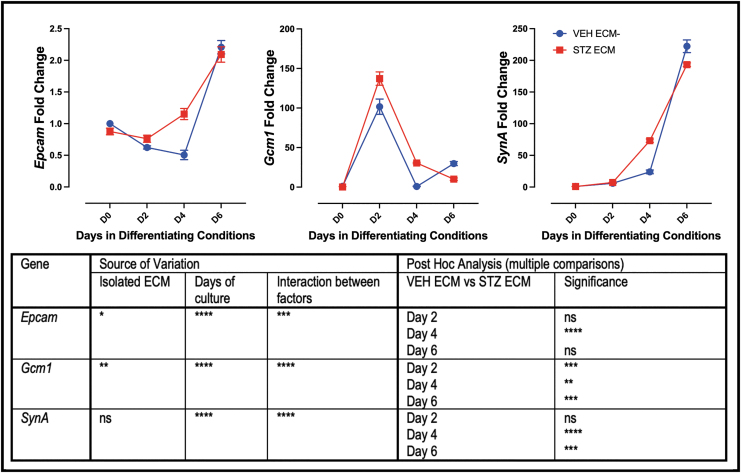
Pathological ECM increases expression of labyrinth trophoblast markers on day 4 in culture. qPCR analysis of markers of labyrinth trophoblast; All three markers have increased expression on day 4 in mTS cells grown on ECM isolated from STZ placentae; two-way ANOVA table identifying the source of variation (ECM, days in culture, and interaction between factors by *row*); post hoc analysis using Šídák's multiple comparisons test identified which day expression was significantly altered by pathological ECM. *Stars* indicate statistical significance, *P ≤ 0.05, **P ≤ 0.01, ***P ≤ 0.001, ****P ≤ 0.0001. Error bars are SEM.

To assess whether the STZ ECM promoted the expression of markers associated with differentiation, labyrinth markers *Epcam*, *Gcm-1*, and *SynA* were evaluated. Like the undifferentiated analysis, there was a difference in gene expression of all three genes between the mTS cells grown on VEH ECM versus STZ ECM (Fig. 7). Confirming that STZ ECM promoted expression of markers associated with differentiation on day 4 of differentiation, each of the three markers had increased expression on the isolated STZ ECM (Fig. 7). However, by day 6 both SynT markers (*Gcm-1* and *SynA*) had reduced expression on the STZ ECM (Fig. 7). This led to speculation that by day 6, the mTS cells on STZ ECM may have elevated markers of junctional zone subtypes.

#### Genes associated with junctional zone trophoblast

**FIG. 8.**
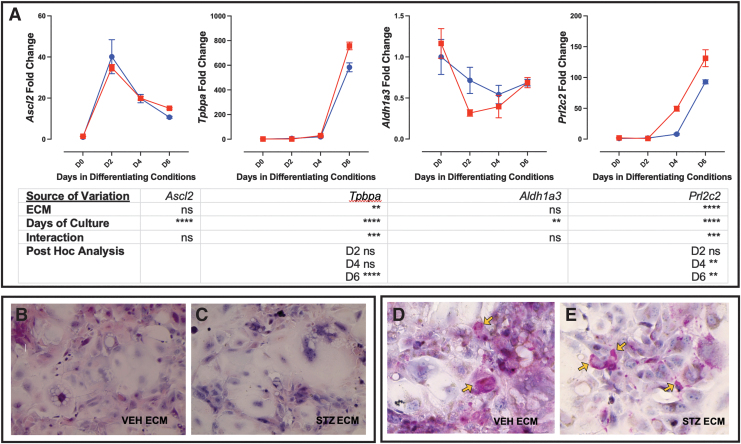
Pathological ECM increases expression of trophoblast markers *Tpbpa* and *Prl2c2*. **(A)** qPCR analysis of markers of junctional zone trophoblast; *Tpbpa* and *Prl2c2* expression peaked on day 6 with increased expression in mTS cells grown on placental ECM isolated from STZ placentae (shown in *red* vs *blue* control); two-way ANOVA table identifying the source of variation (ECM, days in culture, and interaction between factors by *row*); post hoc analysis using Šídák's multiple comparisons test identified which day expression was significantly altered by pathological ECM. **(B)** H&E stain of day 6 mTS cells cultured on placental isolated ECM showing heterogeneous differentiation of the cell cultures; 200 × magnification. **(C)** PAS stain of day 6 mTS cells cultured on placental isolated ECM showing the glycogen accumulation (*orange arrows*) associated with GlyT; 200 × magnification. *Stars* indicate statistical significance, **P ≤ 0.01, ***P ≤ 0.001, ****P ≤ 0.0001. Error bars are SEM. GlyT, glycogen trophoblast cell; PAS, Periodic acid–Schiff.

Supporting STZ ECM increasing markers of junctional zone subtypes, both *Tpbpa* and *Prl2c2* (TGCs) were elevated (day 6 and day 4 and 6, respectively; Fig. 8A). However, expression of *Ascl2* (junctional zone progenitor) and *Aldh1a3* (GlyT) were not altered based on VEH or STZ ECM (Fig. 8A). To confirm differentiation of the mTS cells on placental isolated ECM, Hematoxylin and Eosin (H&E) staining showed that the TS cells grown on the isolated VEH and STZ placental ECM had the heterogeneous morphology of differentiated trophoblast (Fig. 8B, C) [26,50,51]. Furthermore, PAS staining identified the glycogen accumulation typical of GlyT [26,52] in mTS cells grown on VEH or STZ ECM (Fig. 8D, E).

### SynTII population is reduced in STZ placentae

**FIG. 9.**
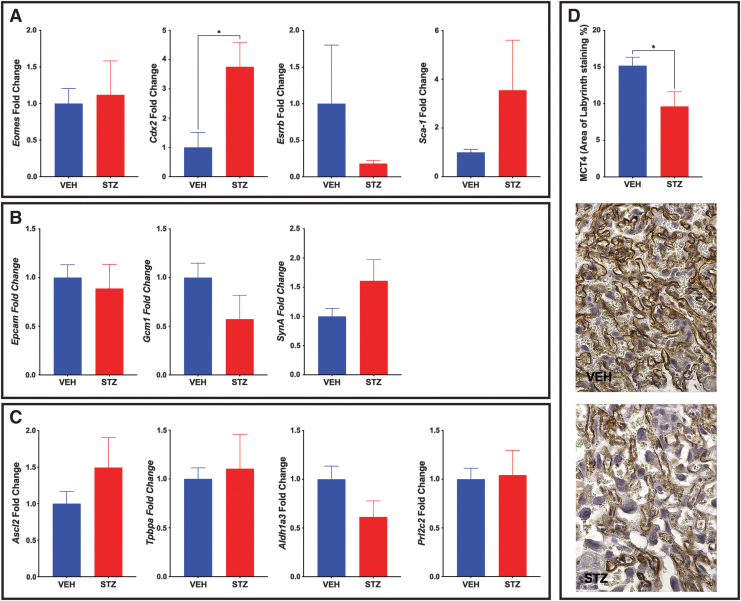
In vivo analysis of trophoblast populations reveals a reduced SynTII population in STZ placentae. **(A)** qPCR analysis of markers of mTS cells; Cdx2 expression is increased in STZ placentae when compared with the VEH control. **(B)** qPCR analysis of markers of labyrinth trophoblast. **(C)** qPCR analysis of markers of junctional zone trophoblast. **(D)** Labyrinth SynTII were reduced in STZ placentae as identified by MCT4 staining at E14.5 with representative histological images. *t*-Test analysis was performed to identify significance. *Stars* indicate statistical significance between the treatment groups, *P ≤ 0.05. Error bars are SEM. Image magnification = 400 × (*n* ≥ 6 placentae per treatment). SynTII, syncytiotrophoblast II; VEH, vehicle.

mTS cells cultured on the ECM isolated from the STZ placentae had altered differentiation profile when compared with the mTS cells cultured on ECM isolated from VEH placentae. Therefore, the expression profile of mTS cell markers and differentiated trophoblast markers in the STZ/VEH placentae were assessed by qPCR. While stem cell markers would typically be expected to be low at E14.5, compromised differentiation might be associated with increased expression of undifferentiated markers. The reduced expression of TS cell markers, *Eomes*, *Cdx2*, and *Esrrb*, in mTS cells grown on isolated STZ ECM did not completely match the in vivo findings. Specifically, *Eomes* was unchanged in vivo, *Cdx2* expression was significantly increased, and *Esrrb*, while decreased, was not statistically significant (Fig. 9A). *Sca-1* was significantly increased in mTS cells grown on isolated STZ ECM, and while expression was increased in vivo, like *Esrrb*, the results were not significant.

In vivo, the expression of labyrinth progenitor marker *Epcam* was unchanged (Fig. 9B), differing from the increased expression noted in vitro. SynT layer II (SynTII) marker, *Gcm-1,* was nonsignificantly reduced in vivo (Fig. 9B), while in vitro expression was increased on day 4 but decreased on day 6 of differentiation. Conversely, *SynA* was nonsignificantly increased in vivo (Fig. 9B), while in vitro, expression was increased on day 4 but decreased on day 6 of differentiation.

In vivo, junctional zone progenitor marker *Ascl2* and Gly-T marker Aldh1a3 expression were similar to the in vitro expression with no significant change (Fig. 9C). However, *Tpbpa* and *Prl2c2* in vivo results differed from the in vitro results, with no significant change in vivo, despite increased expression in vitro on STZ-isolated ECM (Fig. 9C).

While the in vivo *Gcm-1* results were not significant, they were reduced and showed overlap with the in vitro results. As such, in vivo, staining for the monocarboxylate transporter of lactate MCT4 (SLC16A3) located on the SynTII basal membrane was assessed [53]. MCT4 staining in the STZ placentae was significantly reduced (Fig. 9D). *Aldh1a3* results were similarly nonsignificantly reduced in the STZ placentae, and H&E staining was used to visualize the GlyT population, as the morphology of GlyT and Sp-T are different. While the population was not quantified, the histology suggests that the GlyT population may be smaller in the STZ placentae when compared with the VEH placentae (Supplementary Fig. S2).

## Discussion

Over the last decade, there has been much research investigating tissue-specific stem cell niches [54,55]. Defining these niches is the specific ECM that contributes to the regulation of cell behavior, including cell adhesion, cell proliferation, and cell differentiation [56–60]. When the tissue or organ is damaged, the niche can be disturbed. Therefore, understanding how disruption of the niche impacts stem cell function may contribute to our understanding of disease onset and/or progression.

In the placenta, the ECM comprises multiple components that change throughout gestation as the placenta develops and grows, and additionally, this composition changes based on location within the organ. In the human placenta, the villous ECM is characterized by the presence of laminin, while closer to the maternal interface, fibronectin is the dominant component [61]. Trophoblast cells recognize the ECM change through integrin surface receptors, with a change in ECM affecting trophoblast differentiation [62], which can, in turn, alter invasive or proliferative capacity. As placental ECM has the potential to be altered in the event of placental injury, it is important that trophoblast differentiation potential on individual ECM components be established.

This study aimed to assess the expression of genes associated with undifferentiated and differentiated trophoblast cultured on individual ECM components. We showed that undifferentiated mTS cells had the greatest expression of markers associated with mTS cells on laminin. Undifferentiated trophoblast cells are found most abundantly in early gestation, with cell numbers decreasing as the trophoblast cells differentiate and contribute to placental development. Laminin is highly expressed during the blastocyst implantation window [63], when the undifferentiated trophoblast population is expanding. Furthermore, in the human placenta villi, laminin is in proximity to the undifferentiated cytotrophoblast cells [62]. It has additionally been shown in the mouse that in the absence of laminin–integrin interactions, mTS cells cannot expand their population in vivo [6]. Our culture results, together with published in vivo studies, support a role for laminin in maintaining an undifferentiated trophoblast state, although integrin expression was not explored. The migration potential of trophoblast on ECM is well studied, and as such, this study did not include any migration analysis.

Beyond the scope of this study, but worthy of exploring would be whether the individual components alter stem cell behavior. Specifically, stem cell niches can regulate the balance between quiescence, self-renewal, and differentiation [60,64]. Thus, whether the individual components altered the metabolic state of the undifferentiated mTS cells may prove informative when trying to unravel the specifics of the TS cell niche. In the context of mTS cell culture, it is worth noting that the expression of all undifferentiated TS cell markers was maintained, although at a lower level, on the other ECM components. Therefore, studies with the goal of exploring the TS cell niche and looking at mTS cells in their least differentiated state may consider culture on laminin.

Analysis of markers associated with differentiated trophoblast identified that except for the junctional zone progenitor marker *Ascl2* and SynTII marker *Gcm-*1, all other markers evaluated had peak expression on collagen IV. Worth noting is that peak expression on collagen was consistently associated with day 6 peak expression. As collagen IV is found in both the labyrinth and the junctional zone [12] of the mouse placenta, it is not surprising that markers of cell types of both the junctional zone (*Tpbpa*, *Aldh1a3*, and *Prl2c2*) and labyrinth (*SynA*) had increased expression on collagen IV. In the human placenta, collagen IV is associated with the basement membrane and the EVTs [65]. Furthermore, it is used as part of the culture system for the isolation of human TS cells [2]. *Ascl2* expression (a marker of a junctional zone progenitor population) was elevated on laminin, which, when considered with the markers of undifferentiated mTS cells, supports the idea that this substrate helps maintain the expression of genes associated with cells in a “less-differentiated” state.

Of the genes analyzed, the marker most elevated on fibronectin was *Gcm-1*. In the mouse, the *Gcm-1*-positive SynTII cells of the labyrinth are adjacent to the fibronectin-containing basement membrane of the fetal vessels [10,32]. In the STZ placentae, the SynTII population was reduced, alongside reduced fibronectin. When considered with the reduced *Gcm-1* in the mTS cells cultured on STZ-isolated ECM after 6 days in culture, it suggests that the change in ECM, specifically a reduction in fibronectin, may contribute to the reduced SynTII population. In the human placenta, fibronectin staining is localized to the trophoblastic basement membrane of the early gestation chorionic villi early in gestation (week 7) [64] at the same time as GCM-1 is expressed in the villous cytotrophoblast [66], suggesting that this relationship may also be important in human TS cell differentiation. Notably, ECM within the stem cell niche influences stem cell behavior, and stem cells influence the niche in that they secrete and remodel ECM in response to the signals received [67]; however, the secretion of ECM components was not assessed.

Placental pathology is associated with changes to ECM composition. Specifically, there is a relationship between altered ECM and preeclampsia [68–70], with decreased placental laminin [68–70], the collagen IV to laminin ratio altered [71], and fibronectin reduced around the fetal vessels in the villi [72]. We previously showed in our growth-restricted reduced uteroplacental perfusion pressure (RUPP) mouse placenta study (an animal model of preeclampsia) that collagen was reduced in the RUPP placentae [10], and in a separate study that maternal exposure to Δ9-THC during pregnancy alters the placental fetal blood spaces and vessel-associated expression of collagen IV [11]. A mouse study looking at the long- and short-term effects of placental ECM and fetal development identified modification to the synthesis and turnover of ECM before the changes in placental weight [73]. Furthermore, a study of secreted human placental proteins revealed that those uniquely altered in gestational diabetes mellitus are involved in ECM organization while those altered in intrauterine growth restriction affect collagen and laminin formation [74].

While reports of altered ECM in pathology are numerous, we are unaware of studies assessing whether ECM from the pathological placenta can impact trophoblast differentiation.

Using an STZ-induced diabetes model, we generated placentae with significantly altered ECM in the E14.5 STZ placentae compared with the VEH placentae. Placental pathology included altered junctional zone size, reduced labyrinth proliferation, altered fetal blood space perimeter to area ratio, and reduced αSMA^+^ pericytes. Isolation of the ECM isolated from these placentae confirmed that mTS cells cultured on isolated placental ECM maintain their colony-forming potential and express markers associated with undifferentiated and differentiated TS cells. Furthermore, the expression profile of each marker on isolated ECM mirrors the expression profile of each gene on the identified peak individual ECM component (*Eomes*, *Cdx2*, *Esrrb*, and *Sca-1* peak on day 0; *Ascl2* and *Gcm-1* peak on day 2; *Epcam*, *SynA*, *Tpbpa*, and *Prl2c2* peak on day 6; compare Fig. 1 with Fig. 6A, Fig. 2 with Fig. 7, and Fig. 3 with Fig. 8A). The only marker that did not follow this pattern was *Aldh1a3* (GlyT) with neither individual ECM components nor isolated placental ECM significantly altering expression levels.

Furthermore, the analysis confirmed that pathological ECM alters the mRNA expression profile of markers associated with undifferentiated and differentiated mTS cells. Specifically, ECM isolated from the STZ placenta changed the morphology and cell junctions of the less differentiated mTS cells, reduced the expression of mTS cell markers, and the increased expression of the labyrinth and junctional zone markers, implicating a role for pathological ECM in trophoblast differentiation. While human preeclamptic placenta has been used to generate 3D scaffolds, they were recellularized with embryonic fibroblast cells [12]; thus, as far as we are aware, this is the first time that ECM from pathological placenta has been used to culture mTS cells. The results show that healthy versus pathological placental ECM can significantly alter the expression of undifferentiated TS cell genes and differentiated trophoblast genes.

*Tpbpa* expression was increased in the mTS cells grown on the ECM isolated from the STZ placentae; however, the limited analysis of the STZ placentae identified a smaller junctional zone (made up of *Tpbpa*-positive cells). While these results may seem counterintuitive, the cells grown on the isolated pathological ECM did not maintain the same undifferentiated trophoblast morphology, implicating earlier differentiation. As such, it is possible that the cells in the junctional zone were driven to differentiation at the expense of population expansion, although this was not explored in this study.

The qPCR analysis of the STZ and VEH placentae revealed no significant changes in markers of the junctional zone trophoblast populations. The histological assessment, of the cell populations, in the junctional zone suggests that the nonsignificant reduction in *Aldh1a3* may be associated with a reduced Gly-T distribution in the STZ placentae. However, as *Aldh1a3* was unaltered in the isolated ECM studies, it suggests that microenvironment factors other than the ECM may underlie any changes to Gly-T differentiation. The altered ECM may, however, significantly contribute to changes in migration.

Further studies would need to be performed to tease out whether compromised differentiation or altered migration may underlie the change in junctional zone size and what role ECM may play in the STZ placentae.

In the STZ placentae, the SynTII population was reduced, alongside reduced fibronectin. When considered with the reduced *Gcm-1* in the mTS cells cultured on STZ-isolated ECM after 6 days in culture, it suggests that the change in ECM, specifically a reduction in fibronectin, may contribute to the reduced SynTII population in the STZ placentae. However, it is important to note that it is unknown whether the altered ECM reduced the expansion of the progenitor population at the expense of early differentiation or whether the trophoblast had compromised differentiation. However, as proliferation was reduced in the labyrinth, it is tempting to speculate that reduced proliferation may contribute to the reduced SynTII population. As proliferation of mTS cells on the placental ECM was not assessed, it is unknown whether isolated ECM directly affects proliferation.

Proliferation was reduced in the labyrinth of the STZ placenta at E14.5, suggesting that proliferation may have been altered throughout the placenta (including the junctional zone at a time before collection). qPCR analysis of trophoblast populations at E14.5 identified increased expression of TS cell marker *Cdx2* in the STZ placentae, with no significant change to the remaining TS cell or progenitor markers, which would be the populations typically expected to be proliferating. It is worth noting that proliferation within the pericyte or endothelial populations may also contribute to the reduced proliferation in the STZ placentae.

This study demonstrates that individual ECM components and ECM isolated from VEH and STZ placentae can alter the expression of differentiated trophoblast markers in mTS cells. Furthermore, in vivo analysis suggests that a change in fibronectin may underlie compromised SynTII populations. As such, further investigation in the STZ model of diabetes correlating changes in ECM composition and trophoblast differentiation may be warranted. As, several populations of terminally differentiated trophoblast migrate and secrete hormones, cell migration and cellular function analysis, including hormone secretion, would help further define how placental isolated ECM may alter trophoblast function.

Thus, using placental ECM to culture trophoblast and other placental cell populations may prove a useful tool for cell culture. ECM isolation has been shown to yield a solubilized matrix that retains its complex biochemical cues and preserves the collagens, fibronectins, glycoproteins, and heparan sulfates [38]. This assessment was limited in that it only identified whether isolated ECM altered the differentiation profile of mTS cells; therefore, future studies warrant the proteomic characterization of the ECM by mass spectrometry, including MALDI and LC-MS and immunoblotting through SDS–polyacrylamide gel electrophoresis (SDS-PAGE) to quantify and identify pathology-specific differences between the ECM. Furthermore, it is important to consider that tissue ECM also sequesters and locally releases growth factors [75].

This is the first study to show that isolated ECM from diabetic placentae can impact the differentiation of mTS cells, although whether altered expression was due solely to the composition of the ECM itself or due to the biological cues contained within the ECM requires further exploration, as any change to the growth factors sequestered within the ECM may have contributed to the results.

Additionally, the thickness and concentration of the ECM surface when applied to tissue culture plastic need to be considered. Matrigel studies on BeWo and term cytotrophoblast cells have shown that the thickness of the Matrigel influences cell behavior. Specifically, fusion, invasion, matrix degradation, and self-assembly into 3D are affected by matrix thickness, with the surface thickness affecting the cellular stiffness-sensing mechanism that affects the F-actin organization and the cell spread morphology, and the gene expression profiles of integrins and matrix metalloproteinases [19,39]. Thus, studies involving placental isolated ECM will warrant optimization of substrate thickness depending on the desired analysis. Finally, diabetic placentae have been reported to have dysregulated matrix metalloproteinases (MMPs; proteinases that digest and modify ECM and surface receptors) [76], increased inflammation [77], and oxidative factors [78] that may contribute to changes within the ECM composition and lead to altered vascular remodeling.

Now that it has been established that the isolated pathological ECM alters the expression of undifferentiated and differentiated trophoblast markers, it will be important to isolate which ECM contributing factors most significantly affect trophoblast differentiation and/or function.

## Supplementary Material

Supplemental data

Supplemental data

Supplemental data
