# Supplementary material for: Extracellular Matrix Influences Gene Expression and Differentiation of Mouse Trophoblast Stem Cells
Source: Stem Cells Dev. 2023 Oct 3;32(19-20):622–37. doi: 10.1089/scd.2022.0290 (PMC10561768; doi:10.1089/scd.2022.0290)
Supplement: Supplemental data [file Supp_TableS1.pdf]

Table S1. qPCR assay ID for all primers used in the study

	Gene	Mouse Prime PCR SYBR Green Assay ID
TS cells	*Cdx2*	qMmuCID0006160
	Eomes	qMmuCID0009039
	Esrrb	qMmuCED0039638
	Ly6a/Sca-1	qMmuCED0003761
Junctional Zone	*Aldh1a3*	qMmuCED0045366
	Ascl2	qMmuCED0001620
	Prl2c2	qMmuCID0061688
	Tpbpa	qMmuCID0007168
Labyrinth	*Epcam*	qMmuCID0039638
	Gcm1	qMmuCID0023712
	SynA	qMmuCED0003216
Cell Junction/Cell Cell Interaction	*Ctnna1*	qMmuCID0021090
	Ctnnb1	qMmuCID0006137
	Cdh1	qMmuCID0005843
	Cdh3	qMmuCID0005838
	Itgb8	qMmuCID0010499
	Lats1	qMmuCID0018848
	Lats2	qMmuCED0004986
	Stk3	qMmuCID0018528
	Stk4	qMmuCID0009084
	Yap1	qMmuCID0005990
Reference	*Ppia*	qMmuCED0041303
	Ywhaz	qMmuCED0027504
