# Supplementary material for: Extracellular Matrix Influences Gene Expression and Differentiation of Mouse Trophoblast Stem Cells
Source: Stem Cells Dev. 2023 Oct 3;32(19-20):622–37. doi: 10.1089/scd.2022.0290 (PMC10561768; doi:10.1089/scd.2022.0290)
Supplement: Supplemental data [file Supp_FigS1.pdf]

Fig. S1. Representative images of VEH and STZ placental layers. PAS staining identifies glycogen accumulation in Gly-T cells. The junctional zone was defined as the region below the P-TGCs separating the fetal junctional zone from the maternal decidua and the region above the fetal labyrinth (outline in orange). The junctional zone of the STZ placenta is smaller than the junctional zone of the VEH placenta. Image magnification 40x (n≥6 placentae per assessment).

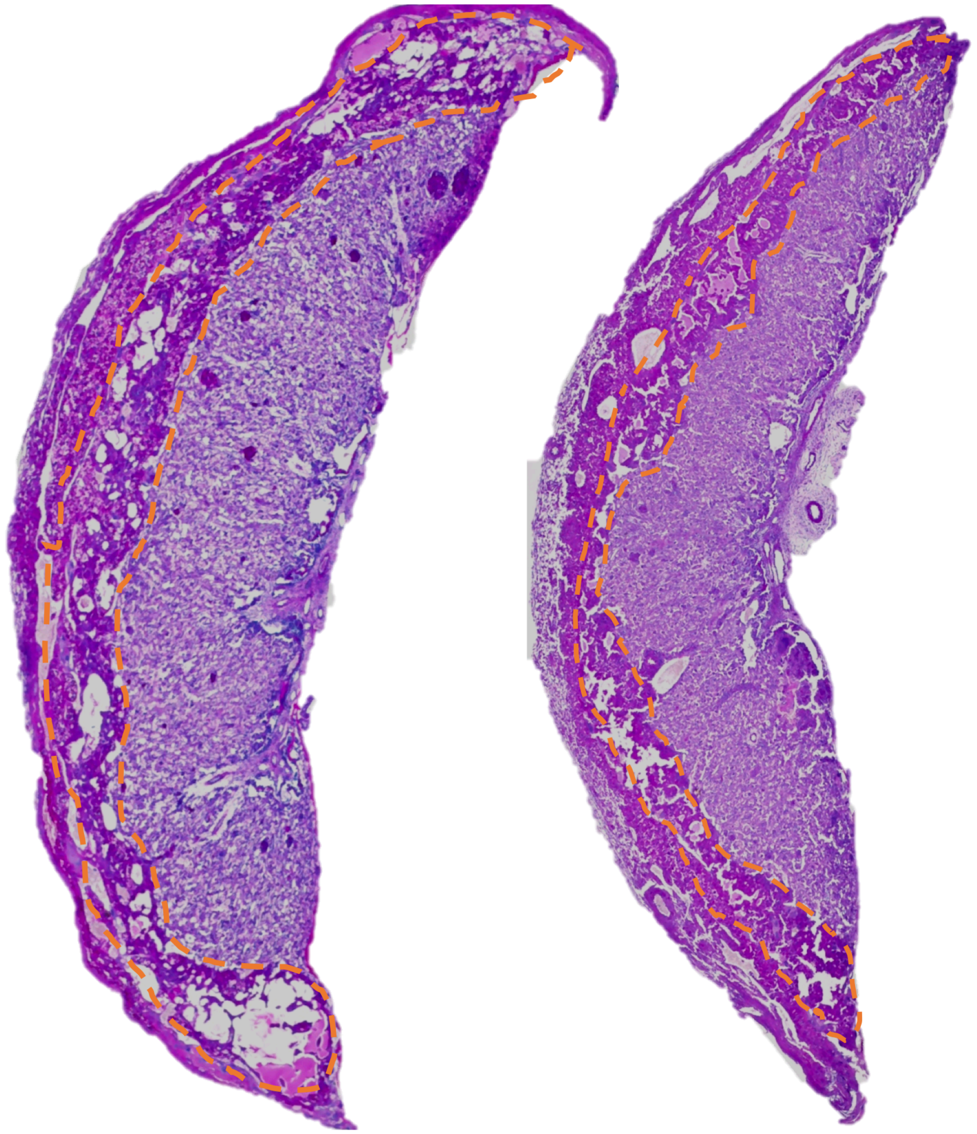

E14.5 VEH Placentae

E14.5 STZ Placentae
