# Supplementary material for: Extracellular Matrix Influences Gene Expression and Differentiation of Mouse Trophoblast Stem Cells
Source: Stem Cells Dev. 2023 Oct 3;32(19-20):622–37. doi: 10.1089/scd.2022.0290 (PMC10561768; doi:10.1089/scd.2022.0290)
Supplement: Supplemental data [file Supp_FigS2.pdf]

Fig. S2. Representative images of VEH and STZ junctional zone trophoblast populations. PAS staining of VEH and STZ placental sections. Red arrow heads identify GlyT cells and green arrows identify Sp-T cells. Image magnification = 400x

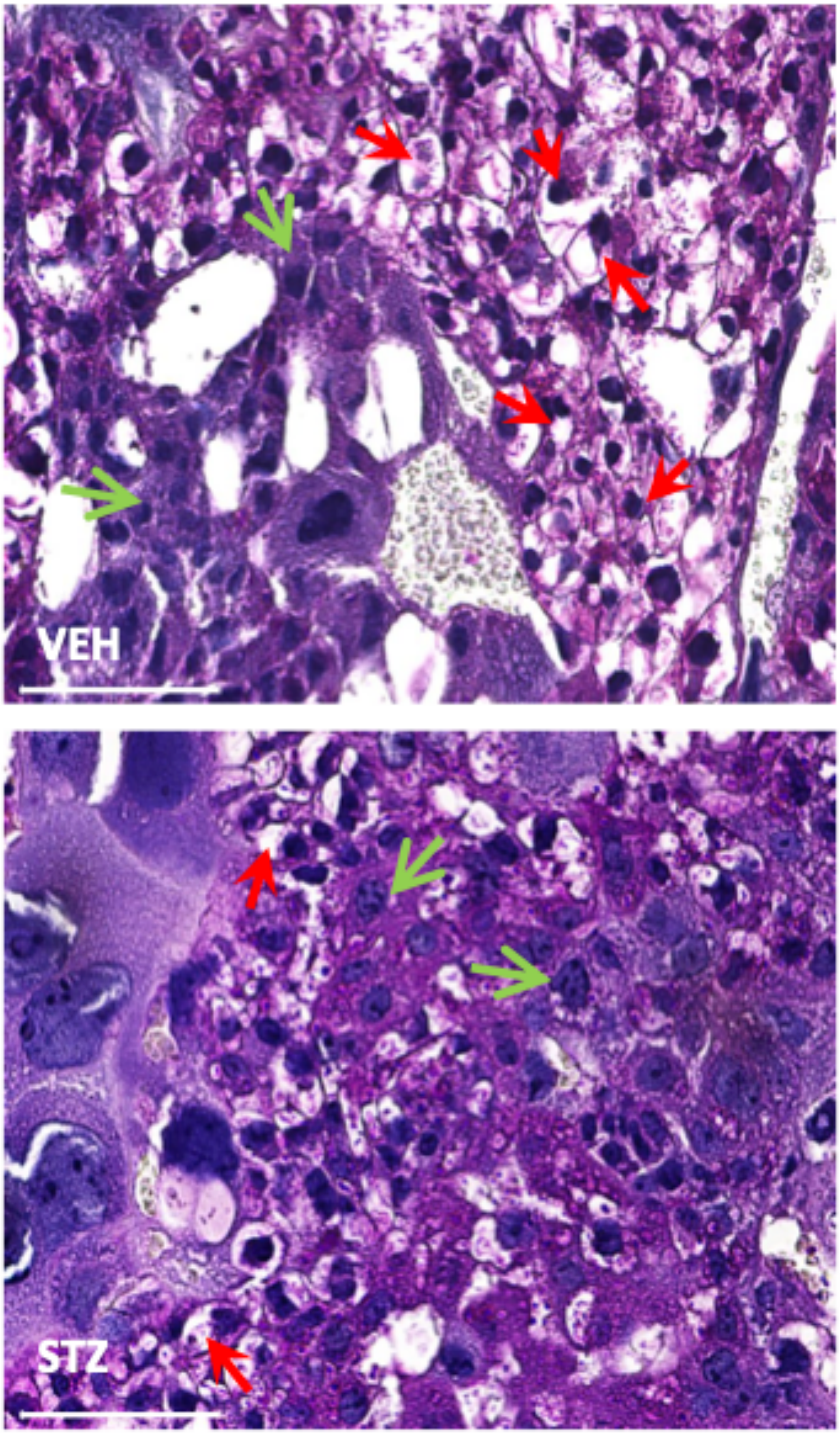

x
